# A *Chlamydia trachomatis* VD1-MOMP vaccine elicits cross-neutralizing and protective antibodies against C/C-related complex serovars

**DOI:** 10.1038/s41541-021-00312-9

**Published:** 2021-04-19

**Authors:** Anja Weinreich Olsen, Ida Rosenkrands, Martin J. Holland, Peter Andersen, Frank Follmann

**Affiliations:** 1 Center for Vaccine Research, Department of Infectious Disease Immunology, Statens Serum Institut, Copenhagen, Denmark; 2 Clinical Research Department, London School of Hygiene & Tropical Medicine, London, United Kingdom; 3 Department of Immunology and Microbiology, University of Copenhagen, Copenhagen, Denmark

**Keywords:** Bacterial infection, Protein vaccines, Bacterial infection

## Abstract

Ocular and urogenital infections with *Chlamydia trachomatis (C.t*.) are caused by a range of different serovars. The first *C.t*. vaccine in clinical development (CTH522/CAF®01) induced neutralizing antibodies directed to the variable domain 4 (VD4) region of major outer membrane protein (MOMP), covering predominantly B and intermediate groups of serovars. The VD1 region of MOMP contains neutralizing B-cell epitopes targeting serovars of the C and C-related complex. Using an immuno-repeat strategy, we extended the VD1 region of SvA and SvJ to include surrounding conserved segments, extVD1^A^ and extVD1^J^, and repeated this region four times. The extVD1^A^*4 was most immunogenic with broad cross-surface and neutralizing reactivity against representative members of the C and C-related complex serovars. Importantly, in vitro results for extVD1^A^*4 translated into in vivo biological effects, demonstrated by in vivo neutralization of SvA and protection/cross-protection against intravaginal challenge with both SvA and the heterologous SvIa strain.

## Introduction

*C.t*. infections cause several human diseases, including trachoma, the leading cause of blindness as a result of infection, and a spectrum of diseases caused by sexually transmitted infections, among them infertility, ectopic pregnancy, and chronic pelvic pain. The infections are caused by a range of serovars separated into 2 major and 2 minor complexes. The serovars of the B complex include B/Ba, D/Da, E, F, L1 and L2, the C complex includes serovars A, C, H, I/Ia, J/Ja, and the two intermediate groups of serovars include F and G/Ga (B-related) and K and L3 (C-related). Serovars A–C, Ba are major causes of trachoma, D–K, Da, Ia, Ja are linked to genital sexually transmitted diseases (STDs), and L1–L3 are commonly associated with lymphogranuloma venereum^1,2^. Vaccine development is ongoing and the ultimate goal is to design vaccines that cover all or the most prevalent serovars.

A *Chlamydia* vaccine, based on neutralizing B-cell epitopes, has been developed in our laboratory with the ability to promote both broadly neutralizing antibodies and high levels of T-cell immunity^3^. The importance of broad serovar coverage became evident in early clinical trials with whole-cell vaccines in both humans and nonhuman primates (NHP). Human volunteers experimentally infected with ocular *C.t*. were protected against rechallenge with a homologous but not against a heterologous serovar^4–6^. NHP vaccination with whole-cell vaccines, NHP studies using sera from ocular infections, and toxicity studies in mice all demonstrated that antibody specificities for different serovars correlated with protection/neutralization against the homologous serovar, but with little cross-reactivity^7–9^.

The main target of surface binding and neutralizing antibodies is the major outer membrane protein (MOMP). MOMP is a transmembrane protein consisting of five constant domains and four surface-exposed variable domains (VDs)^10,11^. Serotype specificity is determined by the *ompA* gene, coding for MOMP, and is located within the four surface-exposed VDs, explaining serological reactivity^12–14^. MOMP is due to its abundance (60% of protein mass) in the outer membrane of the chlamydial elementary body (EB)^15^, an important vaccine target, and has been extensively studied as a vaccine antigen in both its native form (nMOMP)^16–20^ and as recombinant expressed versions (rMOMP)^21–24^. Superior protection of nMOMP has been attributed to strong conformational neutralizing epitopes, which can be difficult to obtain with a recombinantly expressed protein^20^. However, the development of a broadly protective nMOMP vaccine is challenging due to the nature of *C.t*. as an intracellular bacterium and the complicated β-barrel transmembrane structure of MOMP^11,15,25,26^. To address these concerns, our vaccine design strategy is based on selected VDs of MOMP harboring known neutralizing B-cell epitopes.

Antibody responses against VDs during infection have been mapped and characterized by monoclonal antibodies (MAbs)^27–31^. Recognition and neutralization of *C.t*. were either serovar- or serogroup-restricted, and no MOMP-specific MAb had the ability to target or neutralize all serovars. Antibodies directed against the highly conserved TTLNPTIAG sequence in the VD4 region^13,14,31,32^ neutralize B and intermediate groups of serovars^14,31,32^. We previously developed a multivalent vaccine construct Hirep-1 (heterologous immuno-repeat-1), based on VD4s and their surrounding conserved membrane anchors from the most prevalent serovars D–F. To avoid unwanted folding by formation of disulfide bridges, cysteines were exchanged with serines. This vaccine construct demonstrated in vitro and in vivo neutralization and protection against a vaginal challenge with both SvD and SvF^3,33^. A CTH522 vaccine, built on the Hirep concept, has recently completed clinical phase I trial with promising results^34^. Humans vaccinated with CTH522 in combination with either the adjuvants CAF®01 or aluminum hydroxide induced high titers of CTH522-specific antibodies with the functional capacity to in vitro neutralize *C.t*. and induced, in addition, significant levels of CTH522- specific T cells. Neutralizing antibodies from this vaccine has the potential to target the B complex and the intermediate groups of serovars (SvD, E, F, G, K, B, L1, L2, L3), and to a lesser degree the C complex serovars (A, C, H, I, J), indicating different accessibility of this conserved region on the surface of different serovars^14,35^. For C and C-related complex serovars, MAbs directed against the VD1 region have been demonstrated to be effective. Compared to the VD4 region, the VD1 region has a higher degree of serovar-restricted recognition and no VD1-specific MAb has been identified with the ability to target all C/C-related complex serovars^29–31,35^.

Here, we explore the development of a vaccine construct based on the VD1 region and designed to target ocular and genital serovars from C complex serovars. Using our Hirep vaccine design, we produced immuno-repeats of extended regions of VD1 from SvA and SvJ/C, each comprising VD1 regions previously demonstrated to hold neutralizing B-cell epitopes and representing both ocular and genital strains. We compared the immunogenicity and neutralizing activity of the constructs and demonstrated strong cross-neutralizing potential with the VD1 construct from SvA. We finally demonstrated significant protection of extVD1^A^*4/CAF®01vaccinated mice against a vaginal challenge with *C.t*. SvA and SvIa, and we demonstrated a protective role of extVD1^A^*4-specific antibodies in in vivo neutralization experiments challenging mice with *C.t*. SvA pretreated with sera from vaccinated animals.

## Results

### Immunogenicity and neutralizing activity of extended VD1 constructs from SvA and SvJ

With the purpose of generating high titers of functional antibodies against the VD1 region of SvA and SvJ/C, we compared the immunogenicity of different constructs covering the VD1 regions of those serovars. Su et al. previously demonstrated that the VD1 region from *C.t*. SvA was nonimmunogenic in A/J mice and a chimeric peptide composed of a colinear synthesis of the SvA T-cell epitope A8, and the VD1 region was necessary for induction of a VD1-specific antibody response^36^. We used another approach and instead of introducing a T-cell epitope from a distant part of MOMP, we analyzed the effect on the immunogenicity of the VD1 region when extending the VD1 region to cover the surrounding conserved parts. This approach was previously applied to the VD4 region with success^3,33^. Extended versions of the VD1 regions from *C.t*. SvA and SvJ (extVD1^A^ and extVD1^J^) were designed (Table 1). Initially, we compared the immunogenicity of extVD1^A^ with A8-VD1^A^ and VD1^A^ (Supplementary Table 1 for sequences) in CAF01 adjuvant^37,38^. After vaccination, the mice were bled and plasma or sera were tested for IgG reactivity against the VD1^A^ region, the extVD1^A^ region, against intact *C.t*. SvA/HAR-13 and in a neutralization assay, and we found extVD1^A^ to be significantly more immunogenic than A8-VD1^A^ and VD1^A^ alone (Supplementary Fig. 1).

**Table 1:**
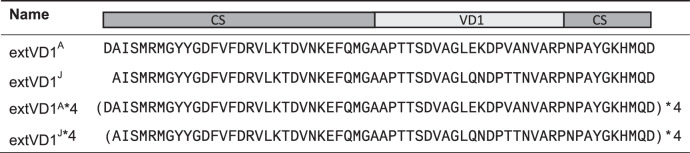
Sequences of VD1 based vaccine constructs.

*CS* conserved segments, *VD1* variable domain 1.

**Fig. 1:**
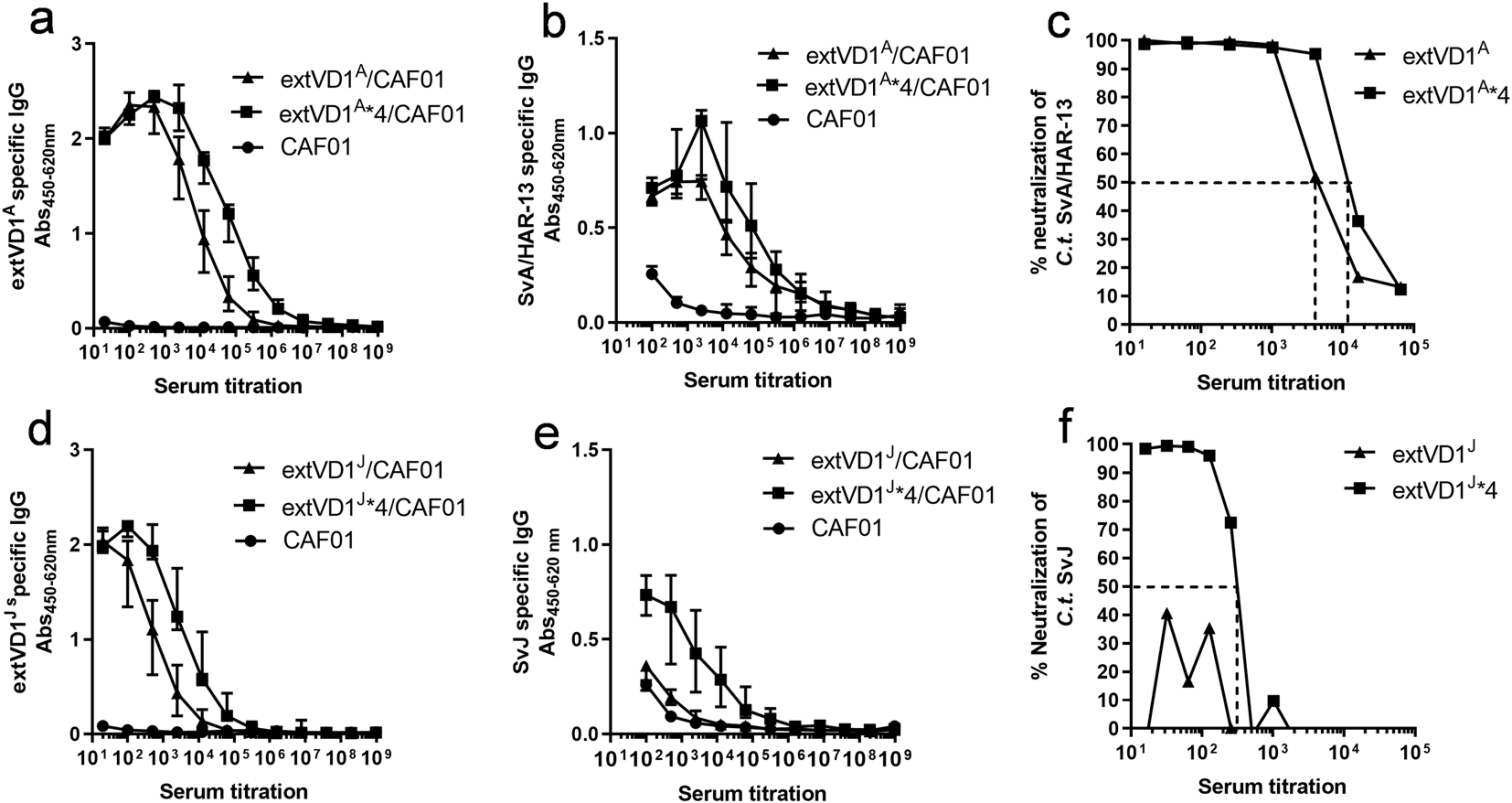
Immunogenicity of monomers (extVD1) and homologous immuno-repeats (extVD1*4). Plasma samples (*n* = 5–6) were isolated 1 week post third subcutaneous (s.c.) vaccination of A/J mice with 10 µg of extVD1^A^, extVD1^J^, extVD1^A^*4, or extVD1^J^*4 emulsified in CAF01, serially diluted and added to extVD1^A^ (**a**), extVD1^J^ (**d**), intact *C.t*. SvA/HAR-13 (**b**), or intact *C.t*. SvJ (**e**) coated plates, and antigen-specific IgG was analyzed by ELISA. Each dot represents the median OD value with 25th and 75th percentiles at each titration step. In vitro neutralization of *C.t*. SvA/HAR-13 (**c**) and *C.t*. SvJ (**f**). Sera isolated 3 weeks post third vaccination were pooled for each group (*n* = 5–6), titrated, mixed with a fixed concentration of bacteria, inoculated onto a HaK cell monolayer, fixed, and inclusions counted. The dotted line indicates the reciprocal NT_50_ titer. The individual experiments were repeated twice with similar results.

Since extending the VD1 region to cover the surrounding conserved parts increased immunogenicity compared to A8-VD1^A^, we continued by designing a recombinant protein based on four repeats of the extVD1^A^ sequence (extVD1^A^*4, Table 1), in order to investigate, if we could further enhance the immune response compared to the monomer as previously published with the extVD4 regions^3^. We further produced an immuno-repeat of extVD1^J^ (extVD1^J^*4, Table 1). The immunogenicity of the two immuno-repeat constructs was compared to their respective monomers (extVD1^A^ and extVD1^J^, Table 1). A/J mice were immunized with 10 µg of the individual constructs in CAF01. After vaccination, the mice were bled and plasma or sera were tested for IgG reactivity against the extVD1 regions (Fig. 1a, d), against intact *C.t*. (Fig. 1b, e) and for functional antibody activity by an in vitro neutralization assay (Fig. 1c, f). The immuno-repeat constructs induced a more than 10 times stronger IgG response compared to the monomers (Fig. 1a, b and Fig. 1d, e)—an IgG response composed of both IgG1 and IgG2a/IgG2b (Supplementary Fig. 2). The IgG response correlated with enhanced ability to neutralize the homologous serovar (Fig. 1c, f). In particular, the SvA immuno-repeat construct induced a very potent neutralizing antibody response with a reciprocal 50% neutralization titer (NT_50_) > 10,000. In comparison, the extVD1^J^*4-specific serum had a weaker ability to neutralize *C.t*. SvJ and a reciprocal NT_50_ titer of around 300 was detected.

### Specificity of the antibody and T-cell responses

**Fig. 2:**
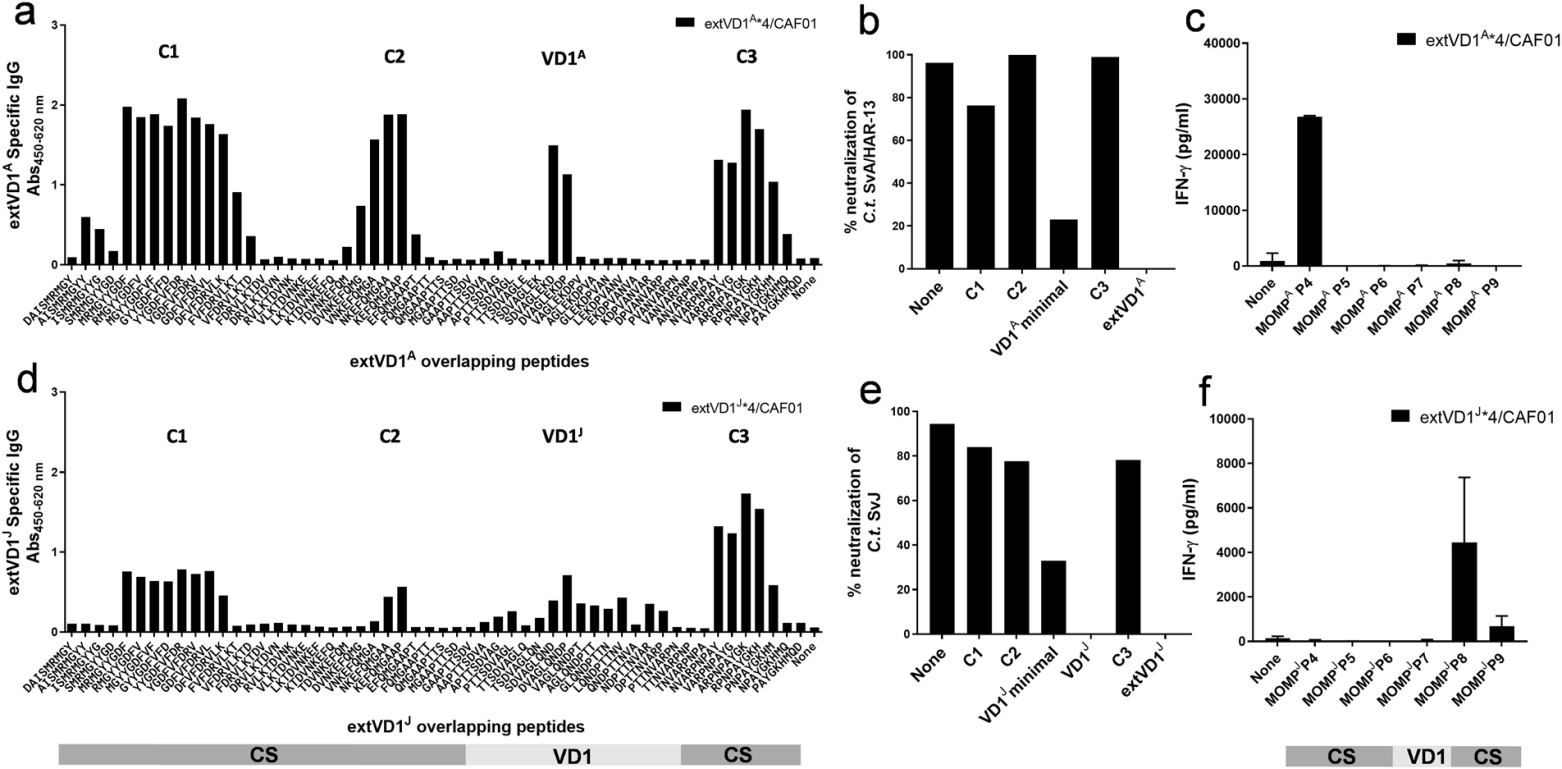
Fine specificity of antibody and T-cell responses after extVD1^A^*4 and extVD1^J^*4 vaccination. A/J mice were immunized 3 times s.c. with either 10 µg of extVD1^A^*4/CAF01 or extVD1^J^*4/CAF01. Three weeks after the last vaccination, sera from immunized mice were pooled (*n* = 5–6), diluted 1:200, and the fine specificity of the IgG antibody responses was studied using a panel of biotinylated overlapping peptides (9-mers with 8 amino acid overlap) representing the extVD1 regions from SvA (**a**) and SvJ (**d**). Each bar represents the mean OD value of two determinations. Competitive peptide inhibition of in vitro neutralization of *C.t*. SvA and SvJ with peptides representing the four identified B-cell epitope regions in extVD1^A^ (**b**) and extVD1^J^ (**e**), respectively. Spleen cells were used to investigate the specific IFN-γ responses using panels of 20–22-mer peptides with 10 amino acid overlap (Supplementary Table 2) spanning extVD1^A^ (**c**) and extVD1^J^ (**f**) regions. Cells from 6 mice/group were pooled and tested in triplicates. Each bar represents mean ± SEM. The individual experiment was repeated 2–3 times with similar results.

For a vaccine to be broadly effective against a range of serovars, it is paramount that B- and T-cell epitopes are located in either conserved regions or that essential binding motifs are conserved among several serovars. To map the region(s) responsible for the neutralization observed after vaccination and to investigate the localization of the T-cell epitopes, we next investigated the specificity of both the B- and T-cell responses using overlapping peptides. Antibody responses were analyzed using 9-mer peptides with 8aa overlap spanning the whole extVD1^A^ and extVD1^J^ regions (Fig. 2a, d). A number of B-cell epitope regions, both within the conserved and specific parts of the constructs, were identified. The extVD1^A^*4 construct induced a response to the previously identified *C.t*. SvA -neutralizing epitope DVAGLEKD (VD1^A^minimal) located in VD1^A ^^31,36^ (Fig. 2a). However, strong antibody responses were also found against three conserved segments C1–C3 (C1: MRMGYYGDFVFDRVLK, C2: VNKEFQMGAAPT, C3: NVARPNPAYGKHM) (Fig. 2a). Likewise, the extVD1^J^ construct induced antibody responses against the variable region (VD1^J^) and the same three conserved segments C1–C3 (Fig. 2d). In contrast to the narrow recognition pattern of VD1^A^, the response to the variable VD1^J^ region was not as well-defined and seemed to cover the entire VD1^J^ region (AAPTTSDVAGLQNDPTTNVARP).

To identify which region(s) were targeted by neutralizing antibodies, we designed peptides representing the conserved (C1–C3), the minimal VD1^A^ epitope DVAGLEKD (VD1^A^ minimal*4), the minimal VD1^J^ epitope DVAGLQND (VD1^J^ minimal*4), the whole VD1^J^ region AAPTTSDVAGLQNDPTTNVARP (VD1^J^), and the extVD1^A^ and extVD1^J^ as positive controls. We performed a competitive inhibition of neutralization assay incubating the serum with and without a high concentration of the individual peptides before incubation with *C.t*. SvA or SvJ. We demonstrated that the sequence/region responsible for generating the major part of the neutralizing antibody response after vaccination, for both constructs, was located in the variable regions (Fig. 2b, e). With the extVD1^A^*4-specific serum, the neutralizing ability was completely inhibited when incubating the serum with the extVD1^A^ peptide and approximately 80% of the ability to neutralize was abrogated when incubating with the minimal VD1^A^ (DVAGLEKD) peptide (Fig. 2b). With the extVD1^J^*4-specific serum, complete inhibition of neutralization was seen when incubating the serum with the whole extVD1^J^*4 sequence. Incubating with the minimal VD1^J^ epitope representing DVAGLQND^31^ reduced the ability to neutralize SvJ by 70%, demonstrating a main role of this region. However, inhibiting with a longer peptide spanning the complete VD1^J^ region AAPTTSDVAGLQNDPTTNVARP, a complete reduction in the neutralizing ability was detected, demonstrating that residues outside the minimal epitope also play a role in the neutralization of SvJ (Fig. 2e).

The specificity of the T-cell response was analyzed by measuring the in vitro stimulatory properties of overlapping 20–22-mer peptides covering the extVD1 regions of MOMP from *C.t*. SvA and SvJ (corresponding to MOMP P4 to MOMP P8, Supplementary Table 2) on splenocytes from vaccinated mice (Fig. 2c, f). The dominant T-cell epitopes were mapped to different regions in the two constructs. For the SvA construct, a dominant T-cell epitope was located in the N-terminal highly conserved part of the construct (MOMP^A^ P4), whereas the T-cell epitope after vaccination with the SvJ construct was located in MOMP^J^ P8 spanning both variable and conserved regions.

### Cross-neutralization of SvA and SvJ

**Fig. 3:**
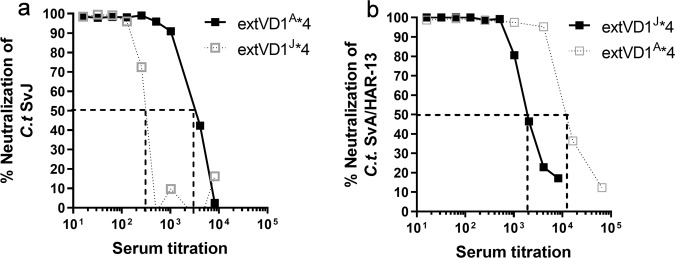
Cross-neutralization of SvA and SvJ with extVD1^J^*4 and extVD1^A^*4-specific sera. A/J mice were immunized with either 10 µg of extVD1^A^*4/CAF01 or extVD1^J^*4/CAF01. Sera were isolated 3 weeks post third s.c. vaccination and pooled for each group (*n* = 5–6), titrated, mixed with a fixed concentration of either *C.t*. SvA or SvJ, and inoculated onto a HaK cell monolayer, fixed, and inclusions counted. Cross-neutralization of *C.t*. SvJ with extVD1^A^*4-specific serum (**a**). For comparison, the homologous neutralization using extVD1^J^*4-specific serum is depicted (dotted gray line). Cross-neutralization of *C.t*. SvA with extVD1^J^*4-specific serum (**b**). For comparison, the homologous neutralization using extVD1^A^*4-specific serum is depicted (dotted gray line). Dotted black lines indicate the reciprocal 50% neutralization titers. The individual experiments have been repeated with similar results.

Since a broad serovar coverage is important, we next investigated if extVD1^A^*4-specific serum could cross-neutralize *C.t*. SvJ and vice versa. ExtVD1^A^*4-specific serum cross-neutralized *C.t*. SvJ with a reciprocal NT_50_ titer of around 3000 (Fig. 3a), a titer that was much higher compared to the titer (NT_50_ = 300) obtained with the homologous extVD1^J^*4-specific serum (dotted curve inserted from Fig. 2f). ExtVD1^J^*4-specific serum was likewise able to cross-neutralize *C.t*. SvA with a reciprocal NT_50_ titer of 2000 (Fig. 3b). This was, however, a much lower neutralization titer compared to the NT_50_ titer of more than 10,000 obtained with the homologous extVD1^A^*4-specific serum (dotted curve inserted from Fig. 2c). Since extVD1^A^*4-specific serum was superior to extVD1^J^*4-generated serum in all investigated aspects of the immune response, we decided to further investigate the biological effects of extVD1^A^*4, i.e., protective efficacy in a vaginal challenge model and the ability to cross-bind and cross-neutralize other *C.t*. serovars.

### In vivo effect of functional antibodies

**Fig. 4:**
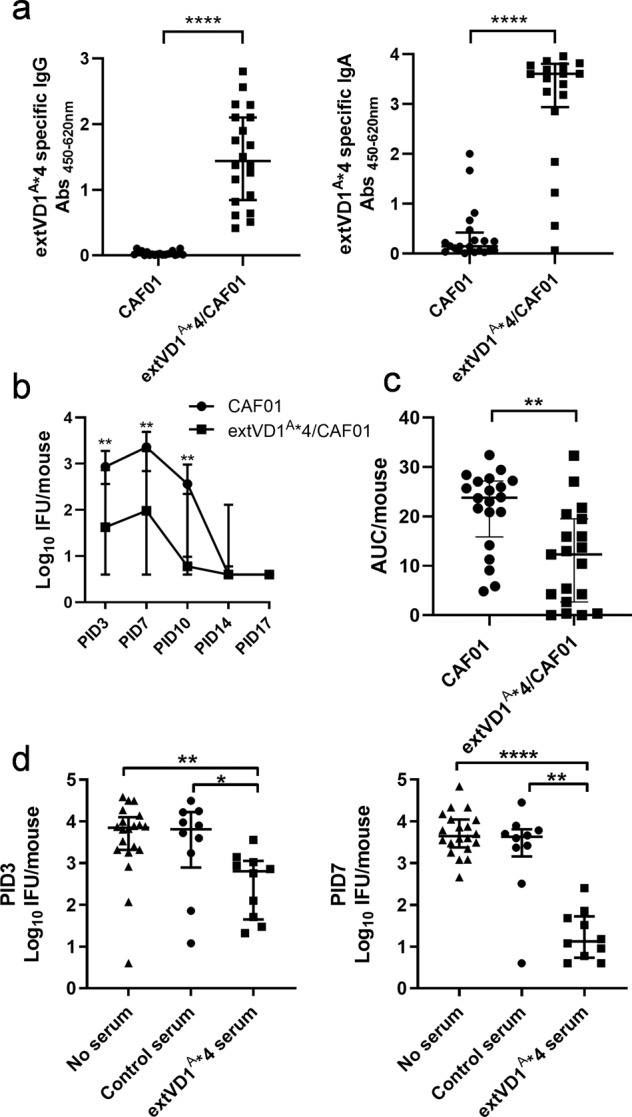
Protective effect of extVD1^A^*4/CAF01 induced immune responses. A/J mice were immunized with extVD1^A^*4/CAF01 or CAF01 alone using the SIM vaccination protocol and the results were pooled from two individual experiments (**a**–**c**). Vaginal wash samples were collected from 20 individual mice/group by flushing the vagina with 100 µl of sterile 1× PBS, diluted 15 times, and extVD1^A^*4-specific IgG and IgA were measured by ELISA (**a**). Each line indicates median level with 25th and 75th percentiles. A Mann–Whitney test was used for comparison among groups ****p < 0.0001. ExtVD1^A^*4/CAF01 (*n* = 19) or CAF01 alone (*n* = 20) vaccinated mice were challenged i.vag. 6 weeks post last vaccination with 1 × 10^6^ IFU/mouse of SvA/HAR-13 (**b**, **c**). Data are presented as median log_10_ IFU with 25th and 75th percentiles recovered from vaginal swabs at day 3, 7, 10, 14, and 17 post infection (PID) (**b**). A Mann–Whitney test was used for comparison among groups. **p < 0.01 at PID3–PID10. Area under the curve (AUC) was calculated and the results presented for individual mice (**c**). Each line represents the median AUC with 25th and 75th percentiles. A Mann–Whitney test was used for comparison among groups **p = 0.0012. In vivo neutralization of SvA with extVD1^A^*4-specific serum (**d**). *C.t*. SvA was incubated with heat-inactivated sera from vaccinated and control mice before i.vag. infection (1 × 10^6^ IFU/mouse) of C3H/HeN mice. In vivo neutralization was assessed by *C.t*. culture at day 3 and 7 post infection. ExtVD1^A^*4 serum, *n* = 10, control serum, *n* = 10, control mice, *n* = 20. Data are presented as log_10_ IFU. Each line represents the median number of IFU with 25th and 75th percentiles. Dunn’s multiple-comparison test was used for comparison among groups. *p < 0.05; **p < 0.01; ****p < 0.0001. The experiment was repeated once with similar results.

To translate the in vitro activity of extVD1^A^*4-specific antibodies into biological activity in vivo, we established a vaginal infection model with *C.t*. SvA in mice. To investigate the in vivo protective efficacy of extVD1^A^*4-specific immune responses, A/J mice were vaccinated three times by the simultaneous (SIM) s.c. and i.n. routes (see “Methods”). Four weeks after the last vaccination, the extVD1^A^*4-specific IgG and IgA antibodies were measured in vaginal wash samples. Statistically significant levels of both isotypes were detected (Fig. 4a). Six weeks after the last vaccination, mice were challenged intravaginally (i.vag) with 1 × 10^6^ inclusion-forming units (IFU) of *C.t*. SvA/HAR-13 and swabbed at consecutive time points post challenge. Mice vaccinated with extVD1^A^*4/CAF01 had significantly reduced chlamydial shedding at post infection day 3 (PID3), PID7, and PID10 (Fig. 4b) (**p < 0.01, at all three time points). Total shedding of IFU from individual mice throughout the infection period (PID3–PID17) in the two groups was compared by measuring the area under the curve (AUC) (Fig. 4c). Significantly less bacteria was shed in the extVD1^A^*4/CAF01-vaccinated group compared to the control group (**p = 0.0012, Mann–Whitney test).

To investigate the functional role of antibodies in the initial phase of infection, independently of an adaptive T-cell response, we performed an in vivo neutralization experiment. Serum was isolated from A/J mice s.c. vaccinated with extVD1^A^*4/CAF01 and diluted 32 times when mixed with *C.t*. SvA/HAR-13. Since C3H/HeN mice are considered to be more susceptible to *C.t*. compared to other mouse strains^39^ and since the MHC haplotype of the mouse is insignificant in this assay, naive C3H/HeN mice were challenged with 10 µl of the mixture (1 × 10^6^ IFU/mouse). Vaginal loads were monitored at PID 3 and 7 (Fig. 4d). Serum from extVD1^A^*4/CAF01-vaccinated mice significantly reduced the ability of *C.t*. to establish a genital tract infection compared to control serum, at both day 3 and 7 post infection, indicating an important role of functional antibodies in controlling infection.

### Cross-recognition of the VD1 regions of *C.t*. with extVD1^A^*4-specific serum

**Fig. 5:**
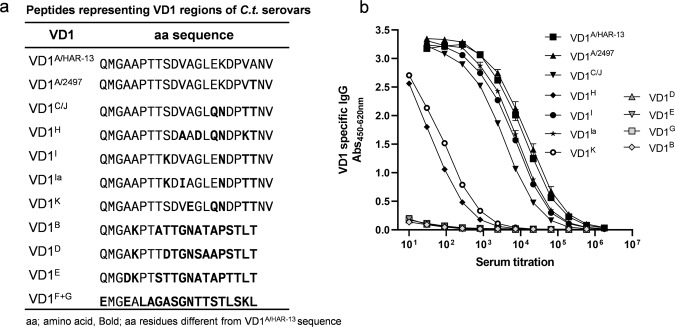
Recognition of peptides representing the VD1 region of *C.t*. serovars with extVD1^A^*4-specific serum. A/J mice were immunized with extVD1^A^*4/CAF01 (*n* = 20) or CAF01 alone (*n* = 20) using the SIM vaccination protocol. Three weeks after the 3rd vaccination, individual serum samples were pooled (*n* = 20), serially diluted in triplicates, and added to peptide-coated plates representing the major part of the VD1 regions of *C.t*. SvA, SvC/J, SvH, SvI, SvIa, SvK, SvB, SvD, SvE, SvF, and SvG. Sequences of VD1 peptides (**a**). Peptide-specific IgG was analyzed by ELISA (**b**). Each dot represents the mean OD value ± SD of triplicate readings at each titration step.

Since the VD1 region plays a dominant role in neutralization of both SvA and SvJ (Fig. 2b, e), we investigated the ability of polyclonal extVD1^A^*4-specific antibodies to recognize 20–22-mer peptides representing the majority of the variable VD1 region of sequence-related serovars (SvA/2497 (clinical isolate), C, H, I, Ia, J, and K) and of more distant serovars (SvD, E, F, G, and B)^13,14^ (Fig. 5a, b). For comparison and as a positive control, we included the corresponding VD1 region from SvA/HAR-13. Strong cross-recognition of the VD1 regions of SvA/2497, C/J, I, and Ia was found, whereas weaker recognition of the VD1 region of SvK and SvH and no recognition of the VD1 region from SvD, E, F, G, and B was detected (Fig. 5b).

### Cross-recognition/neutralization of *C.t*. serovars with extVD1^A^*4-specific serum

The biological effect of antibodies against *C.t*. can roughly be divided into neutralization of infectivity by direct blocking or by facilitating effector functions via complement or effector cells^40–43^. In both cases, a strong recognition of the bacterial surface is a prerequisite for an effector function. Besides the VD1 region, three conserved regions (C1–C3) were strongly recognized (Fig. 2a, d) and could potentially be involved in the surface binding of some serovars. We, therefore, measured the ability of extVD1^A^*4-specific antibodies to recognize the bacterial surface of both C/C-related serovars (SvA/2497, C, H, I, Ia, J, K) but also against B/B-related serovars (SvB, D, E, F, and G) where the VD1 region was not recognized. Pooled serum from extVD1^A^*4-vaccinated mice was analyzed in triplicates in ELISA plates coated with EBs representing the different serovars. ExtVD1^A^*4-specific serum strongly recognized the surfaces of SvA/2497, C, I, Ia, J, and K with reciprocal serum titers giving OD_450–620_ = 0.5 ranging from 15,033 to 151,177. Much lower recognition of SvB, D, E, and F (titers from 32 to 1031) and no recognition of SvG and SvH were detected (Table 2). Except for SvK, this correlated with the VD1 recognition (Fig. 5b). ExtVD1^A^*4-specific serum pools from 2 to 4 experiments were further analyzed for neutralizing ability. Strong reciprocal NT_50_ titers were demonstrated against SvA/2497, C, I, Ia, J, and K ranging from 400 to 10,100, whereas the serum had no neutralizing ability against SvH, B, D, E, F, and G correlating with both weak surface recognition and no VD1 recognition of those serovars (Table 2, Fig. 5b).

**Table 2:** Surface recognition and neutralization of *C.t*. serovars with extVD1^A^*4 specific serum.

*C.t*. serovar	EB specific IgG Mean reciprocal titer at Abs_450–620 nm_ = 0.5	Neutralization Median reciprocal NT_50_ titer
SvA/HAR-13	151,177	11,000
SvA/2497	51,524	10,100
SvC	30,328	6400
SvH	ND	ND
SvI	37,835	2300
SvIa	37,538	400
SvJ	30,159	1800
SvK	15,033	2000
SvB	32	ND
SvD	96	ND
SvE	54	ND
SvF	1031	ND
SvG	ND	ND

*ND* not detectable.

Neutralization titers are the median titer of 2–4 experiments.

**Fig. 6:**
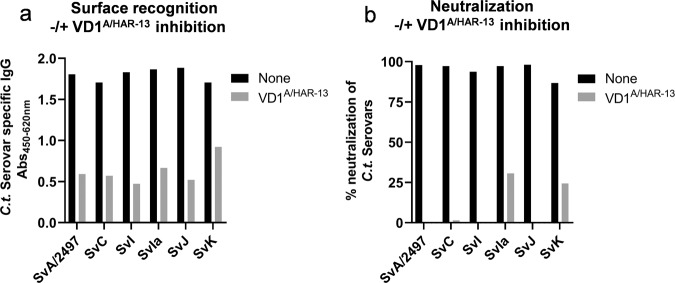
Competitive inhibition of surface recognition and neutralization. A/J mice were immunized with extVD1^A^*4/CAF01 (*n* = 20) or CAF01 alone (*n* = 20) using the SIM vaccination protocol. ExtVD1^A*^4-specific serum was pooled from 20 mice, prediluted, and mixed with 1 mg/ml of VD1^A/HAR-13^ or 1× PBS buffer. After incubation, the mixture was added to *C.t*.-coated ELISA plates in duplicates and *C.t*.-specific IgG measured by ELISA (**a**). Each bar represents the mean of duplicate readings with and without the presence of the VD1^A/HAR-13^ peptide. ExtVD1^A*^4-specific serum was pooled from 20 mice, prediluted, and mixed with 1 mg/ml of VD1^A/HAR-13^ or SPG buffer. After 45 min of incubation, the mixture was further incubated 1:1 with different *C.t*. serovars for 45 min before inoculation onto a HaK cell monolayer, incubated, fixed, and inclusions counted (**b**). Bar represents % neutralization with and without the presence of the VD1^A/HAR-13^ peptide.

To demonstrate that the ability of extVD1^A^*4-specific serum to cross-target other serovars was related to the VD1 region, we competitively inhibited the surface binding by incubating the extVD1^A^*4- specific serum with and without a high concentration of a 22-mer peptide representing the VD1 region of SvA/HAR-13 (VD1^A/HAR-13^ for sequence see Fig. 5a). To ensure that the inhibition of the VD1-specific antibodies was complete in all used serum concentrations, we measured the ability of the VD1^A/HAR-13^ blocked serum to bind to VD1^A/HAR-13^ in an ELISA (Supplementary Fig. 3). The VD1^A/HAR-13^ response was completely blocked since no VD1^A/HAR-13^-specific antibodies were detected. Of significant impact, we found a VD1-independent recognition of the surface of all tested serovars, however, this was most pronounced for SvK (Fig. 6a). This finding could explain the strong surface recognition and neutralization of SvK, despite the lower recognition of the VD1 region, and suggests that regions/amino acids outside the VD1 region are also involved in the surface binding of SvK.

To investigate the impact of the VD1 and the VD1-independent surface recognition on the ability to cross-neutralize, we performed a competitive inhibition of neutralization assay, incubating VD1^A/HAR-13^-blocked extVD1^A^*4-specific serum with the different *C.t*. serovars (Fig. 6b). Incubation of the serum with the peptide led to loss of the major part of the detected neutralization, demonstrating that the VD1 region is responsible for most of the observed cross-neutralization. However, for SvIa and SvK epitopes, amino acid residues outside the VD1 region could play a role for optimal binding of the neutralizing antibodies, since some level of neutralization was still detectable after VD1^A/HAR13^ inhibition.

### Heterologous protection of extVD1^A^*4/CAF01 against a SvIa challenge

**Fig. 7:**
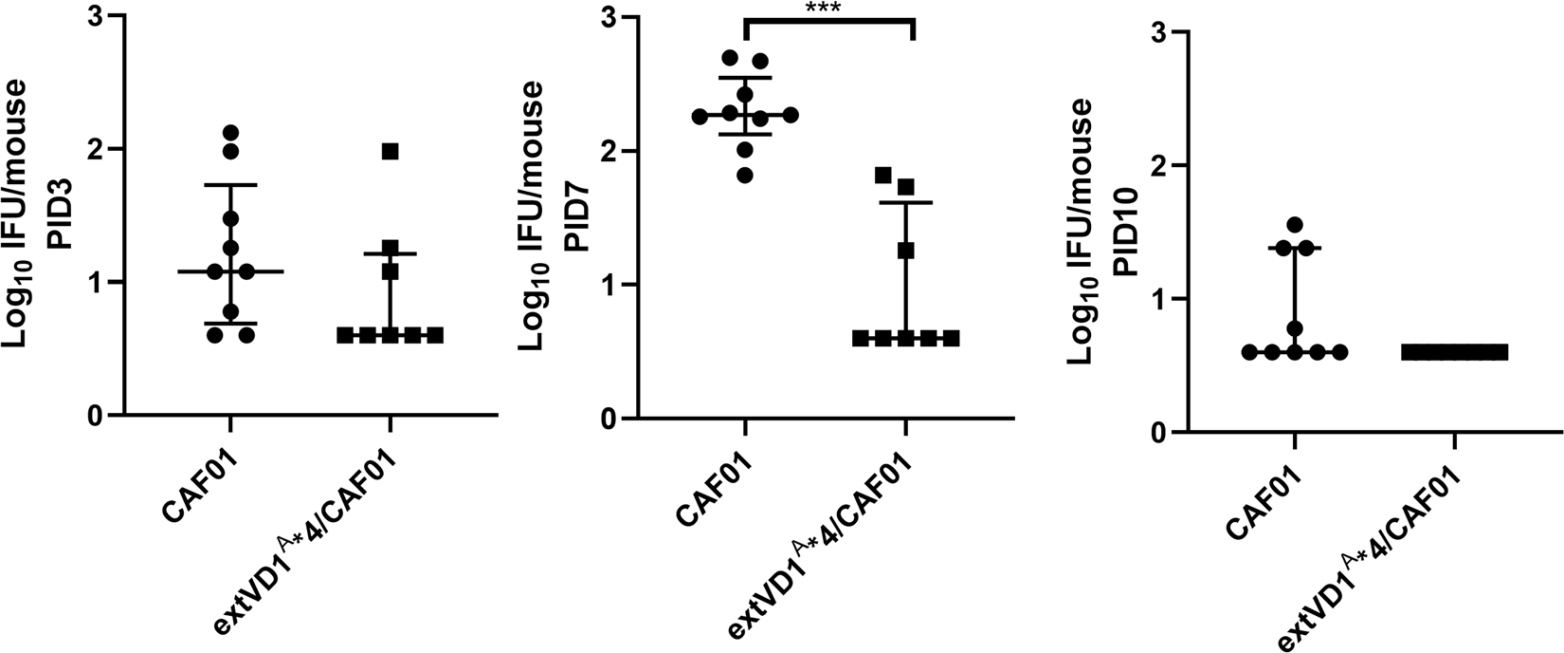
Protective effect of extVD1^A^*4/CAF01 induced immune responses against SvIa challenge. A/J mice were immunized with extVD1^A^*4/CAF01 (*n* = 8) or CAF01 alone (*n* = 9) using the SIM vaccination protocol. Six weeks post last vaccination, the mice were challenged i.vag. with 1 × 10^6^ IFU of SvIa/mouse. Data are presented as log_10_ IFU. Each line represents the median number of IFU with 25th and 75th percentiles recovered from vaginal swabs at days 3, 7, and 10 post infection. A Mann–Whitney test was used for comparison among groups. ***p < 0.001.

Following the demonstration of broad surface recognition and neutralization generated by extVD1^A^*4, we finally investigated if the cross-reactivity of extVD1^A^*4/CAF01-mediated immune responses could be translated into an in vivo effect against a heterologous serovar challenge. SvIa is a prevalent genital serovar of the C complex^44^, and therefore we decided to investigate if extVD1^A^*4/CAF01-vaccinated mice were protected against an i.vag. SvIa challenge. A/J mice were vaccinated three times by the SIM vaccination strategy. Six weeks after the last vaccination, mice were challenged with 1 × 10^6^ IFU of *C.t*. SvIa and swabbed at PID3, 7, and 10 (Fig. 7). Mice vaccinated with extVD1^A^*4/CAF01 had reduced levels of IFU at PID 3, which reached a significant reduction at PID7 (***p < 0.001). At PID10, no bacteria could be detected in vaccinated mice.

## Discussion

*Chlamydia* diseases continue to cause morbidity and there is a need for a broadly protective vaccine covering circulating serovars. The current study focused on developing a vaccine construct with the ability to induce broadly neutralizing antibodies against C/C-related complex serovars (SvA, C, H, I/Ia, J, and K). Exploiting our immuno-repeat vaccine approach^3,33,34^, two novel vaccine constructs, extVD1^A^*4 and extVD1^J^*4, were designed based on the VD1 region of MOMP. Both constructs were highly immunogenic. ExtVD1^A^*4 induced broadly neutralizing antibodies against all tested members of the C/C-related complex, except for SvH. This translated into protective immunity in a mouse genital challenge model of both an ocular (SvA) and a genital (SvIa) strain.

A broad serovar coverage of a *Chlamydia* vaccine is highly preferable, as low serovar coverage could lead to serovar emergence or replacement, as has been observed following vaccination with the Pneumococcal vaccine (PCV-7). Here a steady increase in pneumococcal disease caused by nonvaccine serotypes was reported in some populations^45^. Although a vaccine against the most prevalent sexually transmitted serovars worldwide D–F^46–49^ would have a significant impact, demographical differences have been reported^44,50^. Our CTH522 vaccine, which has recently completed clinical phase I trial^34^, targets the prevalent SvD, E, F, and G with surface-binding and -neutralizing antibodies. With specificities against the VD4 region of MOMP, the CTH522 vaccine can target the B and intermediate groups of serovars, but to a lesser degree the C complex serovars. Therefore, to increase the range of antibody protection against multiple serovars, it would be beneficial to supplement the CTH522 vaccine with constructs that induce VD-specific antibodies with broad C complex serovar recognition.

Since neutralizing MAb antibodies against C/C-related complex serovars have previously been mapped to epitopes in the VD1 region, we took the approach of focusing on the VD1 region of SvA and SvJ/C and improved the immunogenicity of those by extending the VD1 sequence to cover surrounding constant domains (extVD1). We found that molecular repetition of the immunogens further strengthened the immune response by enhancing titers by more than one log_10_ against the protein itself and the bacterial surface. These findings are in agreement with similar studies done in our laboratory on the VD4 region^3^, confirming that even low-valency repeated antigens (2–10) can be superior compared to monovalent antigens^3,51^.

For both extVD1 immuno-repeat constructs (extVD1^A^*4 and extVD1^J^*4), the B-cell epitopes were mapped to four major regions—the VD1 (DVAGLEKD within VD1^A^ and a broader region AAPTTSDVAGLQNDPTTNVARP within VD1^J^) and three conserved regions (C1–C3) (Fig. 2). Strong surface recognition and neutralization were detected with sera from both constructs. Structural reports on MOMP demonstrate accessibility of the VDs on the EB surface. This is in contrast to the constant domains (CDs) adjacent to the VDs that are not predicted to be displayed on the surface of EBs and hence not accessible for antibodies^11,15,22,25,26,52,53^. In support of this, competitive inhibition of neutralization with peptides covering the four B-cell epitope regions demonstrated that antibodies generated against the VD1 regions were responsible for the major part of the observed neutralization of the homologous strain (Fig. 2).

Previous studies investigating the reactivity of VD1-specific MAbs demonstrated that cross-reactivity and neutralization of related serovars were dependent of the ability to bind the VD1 region independently of serovar-specific amino acid residues within the main neutralizing region^29,30^. Analyzing extVD1^A^*4-specific serum, we detected strong cross-binding of the VD1 regions from SvC, I, Ia, J, and the clinical isolate A/2497, lower recognition of VD1^K^ and VD1^H^, and no recognition of the VD1 regions from B/B-related complex serovars (Fig. 5). Except for SvK, a correlation between VD1 reactivity, surface recognition, and neutralization was demonstrated. This was confirmed with a competitive inhibition experiment using the VD1^A/HAR-13^ peptide to compete for recognition. However, for all C/C-related complex serovars, we saw a constant level of surface recognition with serum depleted of VD1^A/HAR-13^-specific antibodies and we speculate that this could be due to antibodies recognizing the conserved regions in close proximity to the VD1 regions (Fig. 6a). Only for SvK and SvIa, this translated into minor non-VD1-specific contributions to neutralization. The exact understanding of this will be the subject for further investigations.

The role of antibodies against *C.t*. is more than direct blocking of infection, i.e., by neutralization. A range of other indirect effector functions like opsonophagocytosis^54^, antibody-dependent complement deposition (ADCD)^55,56^, antibody-dependent cellular cytotoxicity (ADCC)^42^, or combinations thereof could potentially play a role. In this study, we demonstrated an in vivo protective effect of antibodies by preincubating *C.t*. SvA with extVD1^A^*4-specific serum before infecting C3H/HeN mice. This led to a 1–3 log_10_-reduced bacteria level at days 3 and 7 post infection compared to incubation with control serum. Whether this in vivo effect is direct blocking of infection, other antibody-mediated effector functions or a combination is not known. Our results are in line with other studies translating the in vitro neutralization effect of antibodies into in vivo protection, by passive transfer of monoclonal^57,58^, polyclonal antibodies^3,33,59^, or by vaccination^3^. All have generally resulted in reduced early bacterial shedding after a vaginal challenge in mice.

An optimal *Chlamydia* vaccine is composed of a combination of broadly neutralizing B-cell epitopes and conserved T-cell epitopes. An important role of CD4 T cells and IFN-γ has been demonstrated in a range of animal studies^3,60–65^. In humans, CMI and IFN-γ has been associated with a reduced risk of reinfection^66^ and HIV-infected women lacking CD4 T cells have an increased risk of developing *C.t*. pelvic inflammatory disease^67^. Here, we identified T-cell epitopes in extVD1^A^*4 in the conserved N-terminal part of the construct, which is highly preferable in contrast to serovar-specific T-cell epitopes. Of importance in relation to the use of extVD1^A^*4 in a future human vaccine, this region is overlapping a region described by Ortiz et al. to contain human CD4 T-cell epitopes in *C.t*.-infected patients^68,69^.

To evaluate the protective efficacy, we established a SvA/Ia genital infection model in A/J mice. Although *C.t*. SvA is designated an ocular strain, it is well described that ocular serovars can cause genital infections, and vice versa^10,70,71^. We immunized with a combined simultaneous s.c/i.n. (SIM) vaccination strategy previously published by Wern et al.^72^, a strategy that improves mucosal immunity (IgA responses and increased B and CD4 T cells in the genital tract)^72^. We detected strong IgG and IgA responses in the genital tract and found significant protection against an i.vag. challenge with *C.t*. SvA, with a median >1 log reduction in IFU at day 3 and sustained control throughout day 7 and 10 post infection. Whether a combined mucosal and parenteral vaccination strategy is necessary for a future vaccination protocol in humans remains unknown. Our previous studies demonstrated a similar level of protection of SIM and SC vaccination protocols^72^, probably due to high serum IgG levels entering the genital tract in combination with rapid recruitment of circulating Th1/Th17 cells upon challenge^72,73^. In the present study, we saw a highly significant protective effect of serum IgG, when coating the SvA EBs with extVD1^A^*4-specific serum before infection and we have previously seen similar results using VD4-specific neutralizing antibodies^3^ also in adoptive transfer experiments^33^. In humans, IgG is the predominant secreted isotype^74^. However, studies pointing to a protective role of sIgA in humans have been published^75^ and similar observations were found in the minipig model where a correlation between vaginal sIgA correlated with accelerated clearance of *C.t*.^76^. Of significant importance, we also demonstrated protection against i.vag. challenge with a heterologous strain (SvIa), which strengthens the possibility of using this construct to induce broad protection against C/C-related complex serovars. A strong cross-neutralizing antibody response, in combination with induction of conserved T-cell epitopes, could be the key to the observed in vivo protection and cross-protection against SvA/SvIa.

A limitation to the current study is the use of inbred mice in the study of antibody responses. Although antibody responses generally translate well across species, ongoing studies in our lab will investigate responses in outbred species like the guinea pig and NHP, to study if we can induce the same strength and levels of cross-neutralizing antibodies in those species, or if a mixture of multiple serovar-specific immuno-repeats should be considered in a future *C.t*. vaccine. Nonetheless, our studies demonstrate that if strong titers of neutralizing antibodies are obtained by vaccination, an effect on in vivo protection can be seen. Future perspectives are to combine the extVD1^A^*4 with CTH522 in a vaccine that will strengthen both the T-cell responses together with a broad serovar coverage: ocular (SvA, B, and C) and genital (SvD, E, F, G, I, Ia, J, and K) strains. Since the development of vaccines comes with huge investments, this combined ocular/genital vaccine could serve a dual purpose as both STI vaccine and as a vaccine to help with the eradication of trachoma in combination with current control strategies.

## Methods

### Cultivation and harvesting of *C.t.*

*C.t*. SvA/HAR-13, SvC/TW-3, SvH/UW-43/Cx, SvI/UW-12/Ur, SvJ/UW-36/Cx, SvK/UW-31 SvD/UW-3/Cx, SvE/Bour, SvF/IC-Cal-3, SvG/UW-57/Cx (all from ATCC), SvIa/sotonIa3/Ia870 (from the Chlamydia Biobank), and SvA/2497 and SvB/Tunis-864 (from LSHTM) were propagated in Hela 229 cells (ATCC^®^ CCL-2™) or McCoy cells (ATCC® CRL-1696™) and harvested by repeated centrifugation and sonication steps. Finally the bacterial suspension was layered on a 30% renografin solution and centrifuged at 40,000 × *g* for 30 min. After centrifugation, the pellet was resuspended in a sucrose–phosphate–glutamate (SPG) buffer and stored at −80 °C. Serovar typing of the bacteria was confirmed by chromosomal DNA extraction, PCR amplification, and sequencing of the gene and flanking regions of *ompA*. All *C.t*. serovars were tested negative for mycoplasma (Mycoplasma laboratory SSI). IFU of the serovar batches was quantified by titration in McCoy cells. Protein concentrations were determined by bicinchoninic acid protein assay (BCA) (Pierce, Thermo Fisher Scientific, Waltham, Massachusetts, US).

### Antigen cloning and purification

ExtVD1^A^*4 and extVD1^J^*4 were produced as follows. Based on the MOMP amino acid sequences (NCBI YP_328507.1 and AAC31443.1) with addition of six N-terminal histidines, synthetic DNA constructs were codon-optimized for expression in *E. coli* followed by insertion into the pJexpress 411 vector (ATUM). By IPTG, we induced expression in *E. coli* BL-21 (DE3) cells transformed with the synthetic DNA constructs. Inclusion bodies were isolated and extracts were loaded on a HisTrap column (GE Healthcare, Chicago, Illinois, USA), followed by anion exchange chromatography on a HiTrap Q HP column and dialysis to a 20 mM glycine buffer, pH 9.2. Protein concentrations were determined by BCA assay.

### Peptide constructs and overlapping PepSets

The constructs A8-VD1^A^, VD1^A^, extVD1^A^, and extVD1^J^ were produced as synthetic peptides by GeneCust (Boynes, France) (for sequences Table 1 and Supplementary Table 1). Peptides representing C1, C2, and C3, VD1^A^ minimal*4, VD1^J^ minimal*4, VD1^J^ were produced by GeneCust. PepSets of 20–22-mer peptides with 10-aa overlap covering extVD1^A^ and extVD1^J^ and 20–22-mer peptides representing the VD1 regions of the different serovars were produced by GeneCust (Supplementary Table 2 and Fig. 5a). 9-mer (8-aa overlap) biotinylated PepSets covering extVD1^A^ and extVD1^J^ were produced by Mimotopes (Mulgrave, Victoria, Australia) (Fig. 2).

### Animals

Female A/J and C3H/HeN mice, 6–8 weeks of age, were obtained from Envigo, the Netherlands. The mice were housed under standard environmental conditions and provided standard food and water ad libitum. Animal experiments were conducted in accordance with regulations of the Danish Ministry of Justice and animal protection committees by Danish Animal Experiments Inspectorate Permit 2013-15-2934-00978 and 2018-15-0201-01502 and in compliance with European Union Directive 2010/63/EU. The experiments were approved by a local animal protection committee at Statens Serum Institut, IACUC, headed by DVM Kristin Engelhart Illigen.

### Immunization

Mice received a total of three immunizations at two-week intervals either subcutaneously (s.c.) at the base of the tail in a total volume of 200 µl or simultaneously with the intranasal (i.n.) route in a volume of 30 µl. Vaccination protocols were as follows: 3× s.c. (SC vaccination protocol) or 1st s.c., 2nd s.c. + i.n., and 3rd s.c. + i.n (simultaneous (SIM) vaccination protocol). The vaccines given by both routes consisted of 10 µg of antigen, except for the experiment shown in Supplementary Fig. 1, where 25 µg were used for s.c. vaccinations. For s.c. vaccinations, the antigens were diluted in Tris-buffer (pH 7.4) and mixed by vortexing with adjuvant consisting of 50 µg/dose of the glycolipid trehalose 6,6′-dibehenate (TDB) incorporated into 250 µg/dose of cationic liposomes composed of dimethyldioctadecyl-ammonium (DDA) (CAF®01). For the i.n. delivery, the vaccines were delivered without adjuvant. In challenge experiments, the mice were rested 6 weeks before challenge.

### ELISA for antigen-specific antibodies in serum and vaginal washes

Blood was collected after the last vaccination for quantification of vaccine-specific antibodies by enzyme-linked immunosorbant assay (ELISA). Blood was collected from the periorbital vein plexus or the superficial temporal vein into Eppendorf tubes with EDTA (Plasma) or without EDTA (Serum). For isolation of serum, the tubes were centrifuged for 10 min at 10,000 × *g*. To separate plasma, samples were centrifuged for 10 min at 500 × *g*. Vaginal washes were collected by flushing the vagina with 100 µl of sterile 1× PBS and samples stored at −80 °C until analysis. Before dilution, the vaginal wash samples were treated with 25 µg/ml Bromelain (Sigma-Aldrich). Maxisorb plates (Nunc, Roskilde, Denmark) were coated with 50 µl of recombinant antigens (1 µg/ml), peptides (10 µg/ml), or *C.t*. serovars (10 µg/ml) overnight at 4 °C followed by blocking for 2 h in 1× PBS with 2% BSA for IgG and 1× PBS with 1% skimmed milk powder and 0.05% Tween for IgA. The plasma, serum, and vaginal wash samples were serially diluted in 1× PBS with 1% BSA for IgG or 1× PBS with 1% skimmed milk powder and 0.05% Tween for IgA before being added to coated microtiter plates. After washing, HRP-conjugated rabbit anti-mouse IgG (Invitrogen #61-6520), HRP-conjugated goat anti-mouse IgG1 (Southern Biotech #1070-05), HRP-conjugated rabbit anti-mouse IgG2a (Life Technologies #610220), HRP-conjugated goat anti-mouse IgG2b (Invitrogen #M32407), or biotinylated goat anti-mouse IgA (Southern Biotech #1040-08) was added. After 1 h of incubation, the IgA plates were added Streptavidin-HRP (BD Biosciences # 554066). For all isotypes/subtypes, antigen-specific antibodies were detected using TMB-PLUS (Kem-En-TEC, Taastrup, Denmark). The reaction was stopped with H_2_SO_4_ and OD (450–620 nm) was read using an ELISA reader. The results were presented either as titration curves, as absorbance (Abs) at one dilution, or serum titers were reported as the reciprocal of the dilution giving an Abs = 0.5 after subtraction of control serum.

Reactivity against 9-mer overlapping biotinylated Pepsets was investigated by ELISA. Plates were coated with streptavidin, incubated with biotinylated peptides, blocked with skimmed milk powder, washed, and then followed the ELISA procedure described above.

### *C.t*.-specific cellular responses

Splenocytes were isolated from four mice/group 3 weeks post last vaccination, and single-cell suspensions were prepared in RPMI 1640 (Gibco) supplemented with 1% (vol/vol) L-glutamine, 1% nonessential amino acids, 1% sodium pyruvate, 50 µM 2-mercaptoethanol, 1% penicillin–streptomycin, 1% HEPES, and 10% heat-inactivated fetal bovine serum (Biowest, WWR) from pools of 4 mice/group. Cultures were adjusted to 2 × 10^5^ cells/well and stimulated in triplicates with peptides spanning the extVD1 region of *C.t*. SvA or SvJ at a final concentration of 5 µg/ml. Concanavalin A (Con A) was included as a T-cell mitogen for cell viability. After 72 h of incubation, the culture supernatants were harvested, and the amounts of secreted IFN-γ were determined by ELISA. Maxisorb plates (Nunc) were coated with monoclonal rat anti-mouse IFN-γ (BD Pharmingen, #551216) overnight at 4 °C. Plates were blocked with 1× PBS/2% skimmed milk powder. Culture supernatants were diluted in 1× PBS/2%BSA and tested in triplicates. rIFN-γ (BD Pharmingen recombinant Mouse IFN-γ #554587) was used as standard. After 2 h of incubation at room temperature, IFN-γ was detected by biotin-labeled rat anti-mouse IFN-γ (BD Pharmingen #554410) followed by Streptavidin-HRP both diluted in 1× PBS/1%BSA. IFN-γ was detected using TMB-PLUS (Kem-En-TEC), the reaction was stopped with H_2_SO_4_, and OD (450–620 nm) was read using an ELISA reader.

### Neutralization assay

#### In vitro neutralization assay

Hamster kidney cells (HaK) (ATCC® CCL-15) were maintained in RPMI 1640 supplemented with 1% (vol/vol) L-glutamine, 1% nonessential amino acids, 1% sodium pyruvate, 70 µM 2-mercaptoethanol, 10 µg/ml gentamicin, 1% HEPES, and 5% heat-inactivated fetal bovine serum at 37 °C, 5% CO_2_. Cells were grown to confluence in 96-well flat-bottom microtiter plates (Costar, Corning, NY, USA). The different *C.t*. stocks were diluted to a predetermined concentration in SPG buffer and mixed with heat-inactivated (56 °C for 30 min) and serially diluted serum. The mixture was incubated for 45 min at 37 °C and inoculated onto HaK cells in duplicates. After 2 h of incubation at 36 °C on a rocking table, the cells were washed once with RPMI 1640 and further incubated 24 h at 37 °C, 5% CO_2_ in culture media containing 0.5% glucose and cycloheximide (1 µg/ml). The cells were fixed with 96% ethanol and inclusions were visualized by staining with polyclonal rabbit anti-rCT043 serum (produced in our lab), followed by Alexa 488-conjugated goat anti-rabbit immunoglobulin (1:500–1:1000) (Invitrogen #A11008). Cell staining was done with Propidium Iodide (Invitrogen). IFU was enumerated by fluorescence microscopy using an automated cell imaging system (ImageXpress Pico automated Cell imaging system (Molecular Devices, San Jose, California, USA)) counting 25% of each well or by manual counting. The results were calculated as percentage reduction in mean IFU relative to control serum. A serum dilution giving a 50% or greater reduction in IFU relative to the control was defined as neutralizing. The serum dilution giving a 50% reduction in IFU was named reciprocal 50% neutralization titer (NT_50_).

#### In vivo neutralization

*C.t*. SvA was incubated with heat-inactivated and sterile-filtered 32 times diluted serum from extVD1^A^*4/CAF01 s.c.-vaccinated A/J mice or serum from adjuvant control mice. After 30 min at 37 °C, depo-provera-treated C3H/HeN mice were infected with 10 µl of the inoculum (a total of 1 × 10^6^ IFU/mouse), swabbed at days 3 and 7 post infection, and IFU was determined as described in “Vaginal challenge and cultures”.

### Competitive inhibition of surface recognition and neutralization

In the competitive inhibition of surface recognition assay, extVD1^A^*4-specific and control serum (pool of sera from 20 mice) were preincubated for 45 min at 37 °C with 1 mg/ml of the VD1^A/HAR-13^ peptide diluted in 1× PBS or 1× PBS alone. Depending on the serovar being tested, predetermined dilutions of the extVD1^A^*4-specific serum were used to ensure a response within the linear range of the titration curve (SvI/J: 7000×, SvC/Ia: 5000×, and SvK: 2500×). After incubation, the mixture was added to *C.t*.-coated ELISA plates in duplicates and *C.t*.-specific IgG measured by ELISA as described in “ELISA for antigen-specific antibodies in serum and vaginal washes”.

In the competitive inhibition of neutralization assay, extVD1^A^*4-specific serum, extVD1^J^*4-specific serum, and control serum (pool of sera from 20 mice) were preincubated for 45 min at 37 °C with 1 mg/ml of peptides (C1, C2, C3, VD1^A^minimal*4, VD1^A^, extVD1^A^, VD1^J^minimal*4, and VD1^J^ or extVD1^J^) diluted in SPG buffer or SPG buffer alone prior to 45 min of incubation at 37 °C with the different *C.t*. serovars. Depending on the serovar being tested, predetermined dilutions of the extVD1^A^*4-specific serum were used when mixed with the VD1^A/HAR-13^ peptide to ensure between 80 and 100% neutralization in the control wells (SvA/2497:1500×, SvC:500×, SvI:250×, SvJ/K:200×, and SvIa:50×). The extVD1^J^*4-specific serum was prediluted 200 times when mixed with peptides. The mixtures were inoculated onto a HaK cell monolayer in duplicates. After 2 h of incubation at 36 °C on a rocking table, the cells were washed once with RPMI 1640 and further incubated for 24 h at 37 °C, 5% CO_2_, as described previously. Inclusions were fixed, stained, and counted, and percent neutralization was calculated as described in “Neutralization assay”.

### Vaginal challenge and cultures

Ten and three days before *C.t*. SvA or SvIa challenge, the oestrous cycle was synchronized by injection of 2.5 mg of medroxyprogesteronacetat (Depo-Provera, Pfizer, Ballerup, Denmark), increasing mouse susceptibility to chlamydial infection by prolonging dioestrus. The mice were challenged i.vag. with 1 × 10^6^ *C.t*. SvA/HAR-13 or SvIa in 10 µl of SPG buffer. At different time points post infection (PID3, 7, 10, 14, and 17), the mice were swabbed. Swabs were vortexed with glass beads in 0.6 ml of SPG buffer and stored at –80 °C until analysis. The infectious load was assessed by infecting 48-plate wells seeded with McCoy cells with the swab material undiluted and twofold diluted. Inclusions were visualized by staining with polyclonal rabbit anti-MOMP serum made in our lab, followed by an Alexa 488-conjugated goat anti-rabbit immunoglobulin (1:500–1:1000, Invitrogen #A11008). Background staining was done with Propidium iodide (Invitrogen). IFU was enumerated by fluorescence microscopy either manually or by using an automated cell imaging system (ImageXpress Pico automated Cell imaging system) (Molecular Devices) counting 50% of each well. If no IFU were detected in the counted area, 100% of each well was counted manually. Culture-negative mice were assigned the limit of detection of 4 IFU/mouse representing one IFU in the tested swab material (1/4 of the total swab material).

### Statistical analysis

GraphPad Prism 8.3.0 was used for data handling, analysis, and graphic representation. Statistical analysis of vaginal wash samples and log_10_ IFU was performed using the Mann–Whitney test or Kruskal–Wallis (one-way ANOVA) followed by Dunn’s multiple-comparison test. Statistical analysis of the AUC data was done by the Mann–Whitney test. A *p* value of < 0.05 was considered significant.

### Reporting summary

Further information on research design is available in the Nature Research Reporting Summary linked to this article.

## Supplementary information

Supplementary Information

Reporting Summary

## Data Availability

The authors confirm that all relevant data are included in the paper and available from the corresponding author upon request.

## References

[1] Taylor HR, Burton MJ, Haddad D, West S, Wright H (2014). Trachoma. Lancet.

[2] Abdelsamed H, Peters J, Byrne GI (2013). Genetic variation in *Chlamydia trachomatis* and their hosts: impact on disease severity and tissue tropism. Future Microbiol..

[3] Olsen AW, Follmann F, Erneholm K, Rosenkrands I, Andersen P (2015). Protection against *Chlamydia trachomatis* Infection and upper genital tract pathological changes by vaccine-promoted neutralizing antibodies directed to the VD4 of the major outer membrane protein. J. Infect. Dis..

[4] Dawson C (1966). Experimental inclusion conjunctivitis in man. II. Partial resistance to reinfection. Am. J. Epidemiol..

[5] Grayston JT, Wang SP, Yang YF, Woolridge RL (1962). The effect of trachoma virus vaccine on the course of experimental trachoma infection in blind human volunteers. J. Exp. Med..

[6] Jawetz E, Rose L, Hanna L, Thygeson P (1965). Experimental inclusion conjunctivitis in man: measurements of infectivity and resistance. JAMA.

[7] Barenfanger J, MacDonald AB (1974). The role of immunoglobulin in the neutralization of trachoma infectivity. J. Immunol..

[8] Wang SP, Grayston JT, Alexander ER (1967). Trachoma vaccine studies in monkeys. Am. J. Ophthalmol..

[9] Wang SP, Grayston JT (1988). Micro-immunofluorescence antibody responses to trachoma vaccines. Int. Ophthalmol..

[10] Schachter, J. in *Chlamydia - Intracellular Biology, Pathogenesis, and Immunity* (ed R. S. Stephens) Ch. 6 (1999).

[11] Rodriguez-Maranon MJ, Bush RM, Peterson EM, Schirmer T, de la Maza LM (2002). Prediction of the membrane-spanning beta-strands of the major outer membrane protein of Chlamydia. Protein Sci..

[12] Wang SP, Kuo CC, Barnes RC, Stephens RS, Grayston JT (1985). Immunotyping of *Chlamydia trachomatis* with monoclonal antibodies. J. Infect. Dis..

[13] Yuan Y, Zhang YX, Watkins NG, Caldwell HD (1989). Nucleotide and deduced amino acid sequences for the four variable domains of the major outer membrane proteins of the 15 *Chlamydia trachomatis* serovars. Infect. Immun..

[14] Peterson EM, Cheng X, Markoff BA, Fielder TJ, de la Maza LM (1991). Functional and structural mapping of *Chlamydia trachomatis* species-specific major outer membrane protein epitopes by use of neutralizing monoclonal antibodies. Infect. Immun..

[15] Caldwell HD, Kromhout J, Schachter J (1981). Purification and partial characterization of the major outer membrane protein of *Chlamydia trachomatis*. Infect. Immun..

[16] Pal S, Theodor I, Peterson EM, de la Maza LM (2001). Immunization with the *Chlamydia trachomatis* mouse pneumonitis major outer membrane protein can elicit a protective immune response against a genital challenge. Infect. Immun..

[17] Pal S, Luke CJ, Barbour AG, Peterson EM, de la Maza LM (2003). Immunization with the *Chlamydia trachomatis* major outer membrane protein, using the outer surface protein A of Borrelia burgdorferi as an adjuvant, can induce protection against a chlamydial genital challenge. Vaccine.

[18] Pal S, Peterson EM, de la Maza LM (2005). Vaccination with the *Chlamydia trachomatis* major outer membrane protein can elicit an immune response as protective as that resulting from inoculation with live bacteria. Infect. Immun..

[19] Pal S, Peterson EM, Rappuoli R, Ratti G, de la Maza LM (2006). Immunization with the *Chlamydia trachomatis* major outer membrane protein, using adjuvants developed for human vaccines, can induce partial protection in a mouse model against a genital challenge. Vaccine.

[20] Sun G, Pal S, Weiland J, Peterson EM, de la Maza LM (2009). Protection against an intranasal challenge by vaccines formulated with native and recombinant preparations of the *Chlamydia trachomatis* major outer membrane protein. Vaccine.

[21] Hansen J (2008). Liposome delivery of Chlamydia muridarum major outer membrane protein primes a Th1 response that protects against genital chlamydial infection in a mouse model. J. Infect. Dis..

[22] Tifrea DF, Ralli-Jain P, Pal S, de la Maza LM (2013). Vaccination with the recombinant major outer membrane protein elicits antibodies to the constant domains and induces cross-serovar protection against intranasal challenge with *Chlamydia trachomatis*. Infect. Immun..

[23] Ralli-Jain P, Tifrea D, Cheng C, Pal S, de la Maza LM (2010). Enhancement of the protective efficacy of a *Chlamydia trachomatis* recombinant vaccine by combining systemic and mucosal routes for immunization. Vaccine.

[24] Tifrea DF, Pal S, de la Maza LM (2020). A Recombinant *Chlamydia trachomatis* MOMP vaccine elicits cross-serogroup protection in mice against vaginal shedding and infertility. J. Infect. Dis..

[25] Sun G (2007). Structural and functional analyses of the major outer membrane protein of *Chlamydia trachomatis*. J. Bacteriol..

[26] Feher VA (2013). A 3-dimensional trimeric beta-barrel model for Chlamydia MOMP contains conserved and novel elements of Gram-negative bacterial porins. PLoS ONE.

[27] Zhang YX, Stewart S, Joseph T, Taylor HR, Caldwell HD (1987). Protective monoclonal antibodies recognize epitopes located on the major outer membrane protein of *Chlamydia trachomatis*. J. Immunol..

[28] Pal S, Cheng X, Peterson EM, de la Maza LM (1993). Mapping of a surface-exposed B-cell epitope to the variable sequent 3 of the major outer-membrane protein of *Chlamydia trachomatis*. J. Gen. Microbiol..

[29] Qu Z, Cheng X, de la Maza LM, Peterson EM (1993). Characterization of a neutralizing monoclonal antibody directed at variable domain I of the major outer membrane protein of *Chlamydia trachomatis* C-complex serovars. Infect. Immun..

[30] Zhong G, Berry J, Brunham RC (1994). Antibody recognition of a neutralization epitope on the major outer membrane protein of *Chlamydia trachomatis*. Infect. Immun..

[31] Morrison, R. P., Manning, D. S. & Caldwell, H. D. in *Sexually Transmitted Diseases* Vol. 8 (ed. T.C. Quinn) 57–84 (Raven Press Ltd., 1992).

[32] Su H, Caldwell HD (1993). Immunogenicity of a synthetic oligopeptide corresponding to antigenically common T-helper and B-cell neutralizing epitopes of the major outer membrane protein of *Chlamydia trachomatis*. Vaccine.

[33] Olsen AW, Lorenzen EK, Rosenkrands I, Follmann F, Andersen P (2017). Protective effect of vaccine promoted neutralizing antibodies against the intracellular pathogen *Chlamydia trachomatis*. Front Immunol..

[34] Abraham S (2019). Safety and immunogenicity of the chlamydia vaccine candidate CTH522 adjuvanted with CAF01 liposomes or aluminium hydroxide: a first-in-human, randomised, double-blind, placebo-controlled, phase 1 trial. Lancet Infect. Dis..

[35] Zhang YX, Stewart SJ, Caldwell HD (1989). Protective monoclonal antibodies to *Chlamydia trachomatis* serovar- and serogroup-specific major outer membrane protein determinants. Infect. Immun..

[36] Su H, Caldwell HD (1992). Immunogenicity of a chimeric peptide corresponding to T helper and B cell epitopes of the *Chlamydia trachomatis* major outer membrane protein. J. Exp. Med..

[37] Agger EM (2008). Cationic liposomes formulated with synthetic mycobacterial cordfactor (CAF01): a versatile adjuvant for vaccines with different immunological requirements. PLoS ONE.

[38] Pedersen GK, Andersen P, Christensen D (2018). Immunocorrelates of CAF family adjuvants. Semin Immunol..

[39] Darville T (1997). Mouse strain-dependent variation in the course and outcome of chlamydial genital tract infection is associated with differences in host response. Infect. Immun..

[40] Megran DW, Stiver HG, Peeling R, Maclean IW, Brunham RC (1988). Complement enhancement of neutralizing antibody to the structural proteins of *Chlamydia trachomatis*. J. Infect. Dis..

[41] Yang Z, Tang L, Zhou Z, Zhong G (2016). Neutralizing antichlamydial activity of complement by chlamydia-secreted protease CPAF. Microbes Infect..

[42] Naglak EK, Morrison SG, Morrison RP (2017). Neutrophils are central to antibody-mediated protection against genital Chlamydia. Infect. Immun..

[43] Naglak EK, Morrison SG, Morrison RP (2016). IFNgamma is required for optimal antibody-mediated immunity against genital Chlamydia infection. Infect. Immun..

[44] Geisler WM, Suchland RJ, Stamm WE (2006). Association of *Chlamydia trachomatis* serovar Ia infection with black race in a sexually transmitted diseases clinic patient population in Birmingham, Alabama. Sex. Transm. Dis..

[45] Singleton RJ (2007). Invasive pneumococcal disease caused by nonvaccine serotypes among alaska native children with high levels of 7-valent pneumococcal conjugate vaccine coverage. JAMA.

[46] Bandea CI (2008). *Chlamydia trachomatis* serovars among strains isolated from members of rural indigenous communities and urban populations in Australia. J. Clin. Microbiol..

[47] Hsu MC (2006). Genotyping of *Chlamydia trachomatis* from clinical specimens in Taiwan. J. Med Microbiol.

[48] Lysen M (2004). Characterization of ompA genotypes by sequence analysis of DNA from all detected cases of *Chlamydia trachomatis* infections during 1 year of contact tracing in a Swedish County. J. Clin. Microbiol.

[49] Millman K (2004). Population-based genetic and evolutionary analysis of *Chlamydia trachomatis* urogenital strain variation in the United States. J. Bacteriol..

[50] Workowski KA, Suchland RJ, Pettinger MB, Stamm WE (1992). Association of genital infection with specific *Chlamydia trachomatis* serovars and race. J. Infect. Dis..

[51] Minguet S, Dopfer EP, Schamel WW (2010). Low-valency, but not monovalent, antigens trigger the B-cell antigen receptor (BCR). Int Immunol..

[52] Wang Y (2006). Identification of surface-exposed components of MOMP of *Chlamydia trachomatis* serovar F. Protein Sci..

[53] Findlay HE, McClafferty H, Ashley RH (2005). Surface expression, single-channel analysis and membrane topology of recombinant *Chlamydia trachomatis* major outer membrane protein. BMC Microbiol.

[54] Grasse M, Rosenkrands I, Olsen A, Follmann F, Dietrich J (2018). A flow cytometry-based assay to determine the phagocytic activity of both clinical and nonclinical antibody samples against *Chlamydia trachomatis*. Cytom. A.

[55] Lin JS, Yan LL, Ho Y, Rice PA (1992). Early complement components enhance neutralization of *Chlamydia trachomatis* infectivity by human sera. Infect. Immun..

[56] Lausen M (2020). Analysis of complement deposition and processing on *Chlamydia trachomatis*. Med Microbiol. Immunol..

[57] Cotter TW (1995). Protective efficacy of major outer membrane protein-specific immunoglobulin A (IgA) and IgG monoclonal antibodies in a murine model of *Chlamydia trachomatis* genital tract infection. Infect. Immun..

[58] Pal S, Theodor I, Peterson EM, de la Maza LM (1997). Monoclonal immunoglobulin A antibody to the major outer membrane protein of the *Chlamydia trachomatis* mouse pneumonitis biovar protects mice against a chlamydial genital challenge. Vaccine.

[59] O’Meara CP (2016). Induction of partial immunity in both males and females is sufficient to protect females against sexual transmission of Chlamydia. Mucosal Immunol..

[60] Ramsey KH, Rank RG (1991). Resolution of chlamydial genital infection with antigen-specific T-lymphocyte lines. Infect. Immun..

[61] Perry LL, Feilzer K, Caldwell HD (1997). Immunity to *Chlamydia trachomatis* is mediated by T helper 1 cells through IFN-gamma-dependent and -independent pathways. J. Immunol..

[62] Johansson M, Schon K, Ward M, Lycke N (1997). Studies in knockout mice reveal that anti-chlamydial protection requires TH1 cells producing IFN-gamma: is this true for humans?. Scand. J. Immunol..

[63] Morrison SG, Su H, Caldwell HD, Morrison RP (2000). Immunity to murine *Chlamydia trachomatis* genital tract reinfection involves B cells and CD4(+) T cells but not CD8(+) T cells. Infect. Immun..

[64] Gondek DC, Roan NR, Starnbach MN (2009). T cell responses in the absence of IFN-gamma exacerbate uterine infection with *Chlamydia trachomatis*. J. Immunol..

[65] Gondek DC, Olive AJ, Stary G, Starnbach MN (2012). CD4+ T cells are necessary and sufficient to confer protection against *Chlamydia trachomatis* infection in the murine upper genital tract. J. Immunol..

[66] Cohen CR (2005). Immunoepidemiologic profile of *Chlamydia trachomatis* infection: importance of heat-shock protein 60 and interferon- gamma. J. Infect. Dis..

[67] Kimani J (1996). Risk factors for *Chlamydia trachomatis* pelvic inflammatory disease among sex workers in Nairobi, Kenya. J. Infect. Dis..

[68] Ortiz L (1996). *Chlamydia trachomatis* major outer membrane protein (MOMP) epitopes that activate HLA class II-restricted T cells from infected humans. J. Immunol..

[69] Ortiz L, Angevine M, Kim SK, Watkins D, DeMars R (2000). T-cell epitopes in variable segments of *Chlamydia trachomatis* major outer membrane protein elicit serovar-specific immune responses in infected humans. Infect. Immun..

[70] Ikehata M, Numazaki K, Chiba S (2000). Analysis of *Chlamydia trachomatis* serovars in endocervical specimens derived from pregnant Japanese women. FEMS Immunol. Med. Microbiol..

[71] Lesiak-Markowicz I, Schotta AM, Stockinger H, Stanek G, Markowicz M (2019). *Chlamydia trachomatis* serovars in urogenital and ocular samples collected 2014-2017 from Austrian patients. Sci. Rep..

[72] Wern JE, Sorensen MR, Olsen AW, Andersen P, Follmann F (2017). Simultaneous subcutaneous and intranasal administration of a CAF01-adjuvanted Chlamydia vaccine elicits elevated IgA and protective Th1/Th17 responses in the genital tract. Front Immunol..

[73] Nguyen N (2020). Parenteral vaccination protects against transcervical infection with *Chlamydia trachomatis* and generate tissue-resident T cells post-challenge. NPJ Vaccines.

[74] Fahrbach KM, Malykhina O, Stieh DJ, Hope TJ (2013). Differential binding of IgG and IgA to mucus of the female reproductive tract. PLoS ONE.

[75] Brunham RC, Kuo CC, Cles L, Holmes KK (1983). Correlation of host immune response with quantitative recovery of *Chlamydia trachomatis* from the human endocervix. Infect. Immun..

[76] Lorenzen E (2015). Intramuscular Priming and Intranasal Boosting Induce Strong Genital Immunity Through Secretory IgA in Minipigs Infected with *Chlamydia trachomatis*. Front Immunol..

