# Publisher Correction: A *Chlamydia trachomatis* VD1-MOMP vaccine elicits cross-neutralizing and protective antibodies against C/C-related complex serovars

**DOI:** 10.1038/s41541-022-00521-w

**Published:** 2022-08-30

**Authors:** Anja Weinreich Olsen, Ida Rosenkrands, Martin J. Holland, Peter Andersen, Frank Follmann

**Affiliations:** 1 Center for Vaccine Research, Department of Infectious Disease Immunology, Statens Serum Institut, Copenhagen, Denmark; 2 Clinical Research Department, London School of Hygiene & Tropical Medicine, London, United Kingdom; 3 Department of Immunology and Microbiology, University of Copenhagen, Copenhagen, Denmark

**Keywords:** Bacterial infection, Protein vaccines, Bacterial infection

Correction to: *npj Vaccines* 10.1038/s41541-021-00312-9, published online 19 April 2021

In the original version of this article, Table 2 was missing from the PDF version. The PDF version has now been corrected. The HTML version of this article did not need correcting.

