# Supplementary material for: A Chlamydia trachomatis VD1-MOMP vaccine elicits cross-neutralizing and protective antibodies against C/C-related complex serovars
Source: NPJ Vaccines. 2021 Apr 19;6:58. doi: 10.1038/s41541-021-00312-9 (PMC8055873; doi:10.1038/s41541-021-00312-9)
Supplement: Supplementary file 1 — Supplementary Information [file 41541_2021_312_MOESM1_ESM.pdf]

Supplementary Table 1. Amino acid sequences of VD1 constructs

**Name**	**VD1 constructs**
VD1^{A}	GAAPTTSDVAGLEKDPVANVA
A8-VD1^{A}	ALNIWDRFDVFCTLGATTGYLKGNS-GAAPTTSDVAGLEKDPVANVA
extVD1^{A}	DAISMRMGYYGDFVFDRVLKTDVNKEFQMGAAPTTSDVAGLEKDPVANVARPNPAYGKHMQD

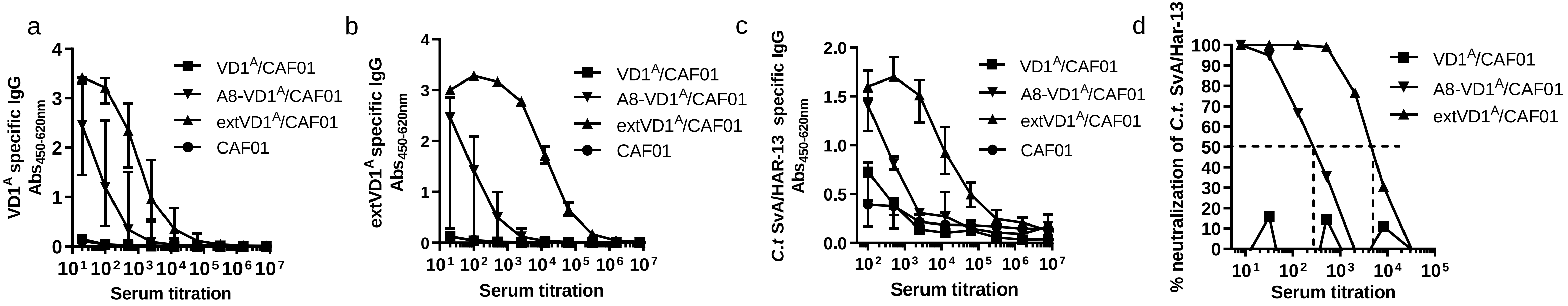

Supplementary Fig. 1. **Comparison of antibody responses and neutralization with different VD1 based constructs**. A/J mice were immunized 3 times with 25 µg of either VD1^{A}, A8-VD1^{A}, or extVD1^{A} emulsified in CAF01 (n=5). After the final vaccination, the mice were bled and plasma were tested for IgG reactivity against VD1^{A} **(a)** against extVD1^{A} **(b)** and against intact *C.t.* SvA/HAR-13 **(c)** by ELISA. Each dot represents the median OD value with 25th and 75th percentiles at each titration step. *In vitro* neutralization of *C.t.* SvA/HAR-13 **(d)**. Sera isolated 3 weeks post third vaccination were pooled for each group (n=5), titrated, mixed with a fixed concentration of bacteria, inoculated onto a HaK cell monolayer, fixed and inclusions counted. The dotted line indicates the reciprocal NT_{50} titer.

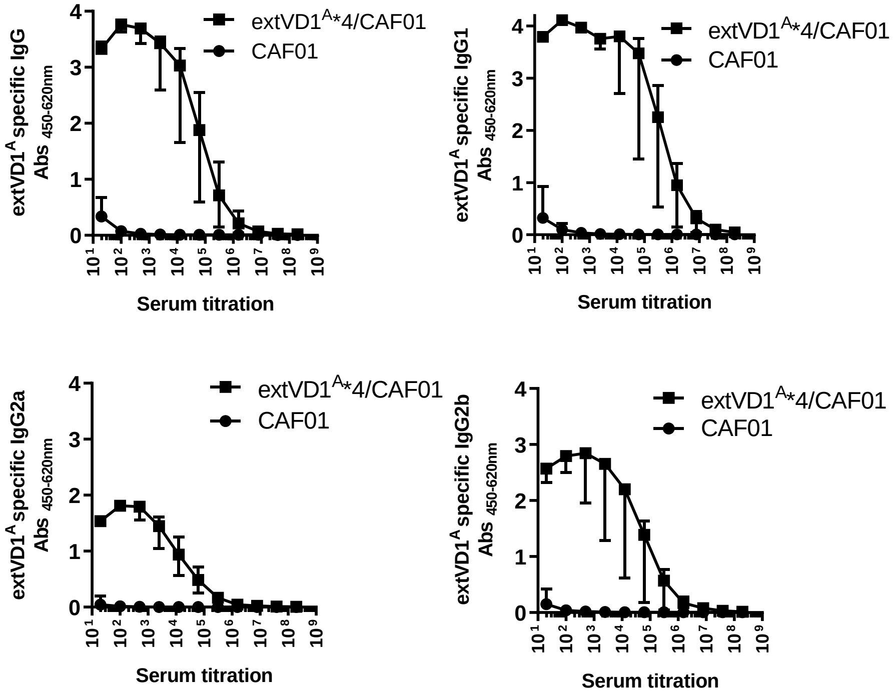

Supplementary Fig. 2. **IgG subtype responses after extVD1^{A}*4/CAF01 vaccination.** A/J mice (n=6) were immunized 3 times SC with 10 µg extVD1^{A}*4 emulsified in CAF01 After the final vaccination, the mice were bled and plasma were tested for IgG, IgG1, IgG2a, and IgG2b reactivity against extVD1^{A} . Each dot represents the median OD value with 25th and 75th percentiles at each titration step.

Supplementary Table 2. Peptides spanning extVD1 of MOMP from SvA and SvJ

**Overlapping peptides P4-P9 covering the extVD1 sequence of MOMP**	**Sequences of 20-22 mer peptides with 10 aa overlap**	
	**SvA**	**SvJ**
MOMP P1-P3	………………………………..........	………………………………..........
**MOMP P4**	*TWCDAISMRMGYYGDFVFDR*	*TWCDAISMRMGYYGDFVFDR*
**MOMP P5**	*GYYGDFVFDRVLKTDVNKEF*	*GYYGDFVFDRVLKTDVNKEF*
**MOMP P6**	*VLKTDVNKEFQMGAAPTTSD*	*VLKTDVNKEFQMGAAPTTSD*
**MOMP P7**	QMGAAPTTSDVAGLEKDPVANV	QMGAAPTTSDVAGLQNDPTTNV
**MOMP P8**	GLEKDPVANVARPNPAYGKH	GLQNDPTTNVARPNPAYGKH
**MOMP P9**	*ARPNPAYGKHMQDAEMFTNA*	*ARPNPAYGKHMQDAEMFTNA*
P10-P36	……………………....................	……………………....................

Italic sequences: identical sequences in SvA and SvJ

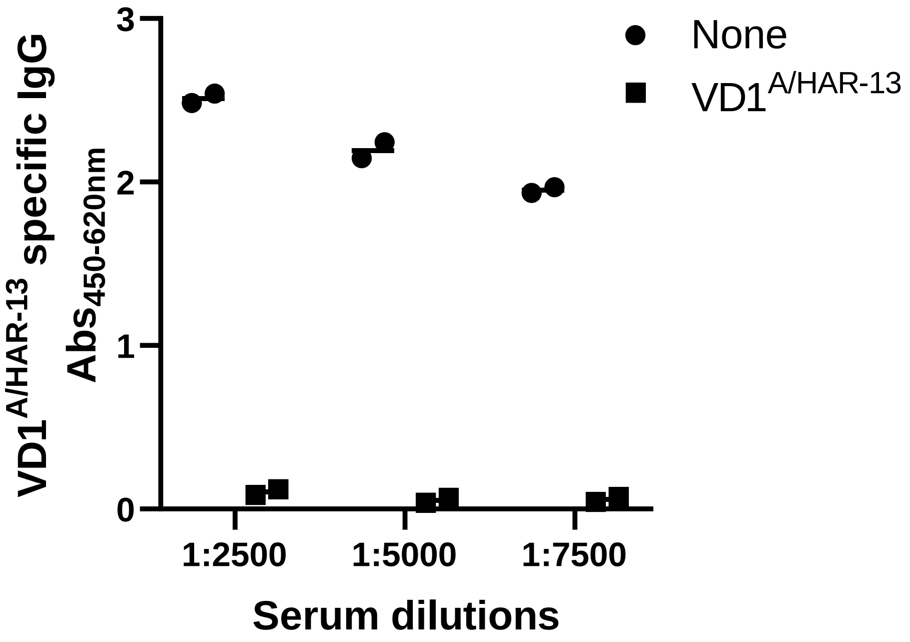

Supplementary Fig. 3. Inhibition of extVD1^{A}*4 specifc serum dilutions with the VD1$^{A/HAR-13}$ peptide. A/J mice were immunized with extVD1^{A}*4/CAF01 and serum harvested 3 weeks after final vaccination. Dilutions of the serum were incubated at 37 °C with 1 mg/ml of VD1$^{A/HAR-13}$ for 45 min. and added in duplicates to VD1$^{A/HAR-13}$ coated plates. VD1$^{A/Har13}$ specific IgG was measured by ELISA. Each dot represents individual readings and line shows the mean. Complete inhibition of VD1 specific antibodies in VD1$^{A/Har-13}$ inhibited serum compared to non-inhibited serum was seen in all tested serum dilutions.
